# Short‐ and Long‐Term Efficacy of Hyperbaric Oxygen Therapy in Irradiated Breast Cancer Patients With Long‐Standing Lymphedema: A Prospective Case Series

**DOI:** 10.1002/cnr2.70109

**Published:** 2025-01-08

**Authors:** Imjai Chitapanarux, Siriarrayapa Chachvarat, Patumrat Sripan, Thienchai Pattarasakulchai, Nuttaya Pattamapaspong, Thanat Kanthawang, Montana Buntragulpoontawee

**Affiliations:** ^1^ Division of Radiation Oncology, Department of Radiology Chiang Mai University Chiang Mai Thailand; ^2^ Nakhon Si Thammarat Cancer Hospital, Nakhon Si Thammarat Nakhon Si Thammarat Thailand; ^3^ Research Institute for Health Sciences Chiang Mai University Chiang Mai Thailand; ^4^ Hyperbaric Oxygen Therapy Center, Chiang Mai University Chiang Mai Thailand; ^5^ Division of Diagnostic Radiology, Department of Radiology Chiang Mai University Chiang Mai Thailand; ^6^ Neuro‐Mobility Unit, Department of Rehabilitation Medicine Chiang Mai University Chiang Mai Thailand

**Keywords:** efficacy, hyperbaric oxygen treatment, long standing, lymphedema

## Abstract

**Background:**

Several studies have explored the advantage of treatment with hyperbaric oxygen (HBO) for upper extremity lymphedema in irradiated breast cancer patients and reported controversial results. This prospective case series aimed to document the short‐ and long‐term efficacy of this therapy, focusing on the arm volume and functional assessment in breast cancer patients with a history of long‐standing lymphedema for more than 2 years.

**Case:**

Six breast cancer patients with long‐standing lymphedema were enrolled. All of them received breast surgery and dissection of axillary lymph nodes, chemotherapy, and postoperative radiotherapy. The average duration from the onset of lymphedema to HBO treatment was 9 ± 5.92 years (range 2–17). All patients were treated for 90 min in the HBO chamber with 100% oxygen at a higher pressure of two times the atmospheric pressure, five times a week for 30 sessions. We measured the circumference of both arms and calculated the arm volume before treatment (baseline), after 30 sessions of treatment, and after treatment at 3, 6, and 24 months. Functional assessment focused on the ability of the arm, shoulder, and hand using the Thai Quick‐DASH questionnaires, which were assessed by the patients at the same periods. The change of arm volume and Thai Quick‐DASH score at each time point were compared to the starting point data before HBO.

**Conclusion:**

The change of arm volume and mean Quick‐DASH score had decreased at 3, 6, and 24 months after treatment compared to the starting point before HBO, However, these changes were not statistically significant.

## 1 Introduction

Giving the latest evidence from online global cancer data in 2018, breast cancer is the highest incidence cancer in Thai women with 35.7 per 100 000 population [1]. Surgery is considered the primary therapy for this cancer. Adjuvant treatments include radiotherapy (RT) and systemic treatment. These multimodality treatments are associated with significant complications. One of the common late complications is upper extremity lymphedema, especially subsequent to axillary lymph node removal and postoperative irradiation. A systematic review of 72 studies [2] found that more than 20% of breast cancer survivors will develop upper limb lymphedema.

A recent prospective observational study [3] from Brazil reported a rising cumulative incidence of lymphedema over time, with rates of 13.5% at 2 years, 30.2% at 5 years, and 41.1% at 10 years.

Upper extremity lymphedema is a late side effect that typically develops from 1 to 5 years after breast cancer treatment. This chronic and progressive condition is characterized by abnormal swelling due to the accumulation of lymph fluid. It often arises from a blockage or interruption of the lymphatic system, which can be associated with earlier surgery or RT [4, 5, 6].

There are multiple factors that are related to the occurrence of acquired lymphedema. According to Park, Lee, and Chung [7], these include advanced cancer stage, modified radical mastectomy, axillary lymphadenectomy, regional nodal irradiation to the axilla area, and high body mass index (BMI). Similarly, Ugur et al. [8] identified the aggressiveness of surgery, the number of axillary lymph nodes removed, postoperative axillary irradiation, patients with obesity, and infections as key risk factors.

The latest evidence‐based review [9] identified additional risk factors, including lack of breast reconstruction, neoadjuvant and adjuvant chemotherapy, subclinical edema, and cellulitis.

This late complication is associated with symptoms such as discomfort, a feeling of heaviness, functional disability, disfiguration, psychological suffering, and an increased risk of recurrent infections. These factors significantly impact the quality of life (QoL) of affected individuals [10].

Treatment of chronic lymphedema is primarily supportive, aiming to avoid worsening and relieve symptoms. According to an international consensus on lymphedema treatment [11], the most effective practices include complete decongestive therapy, special exercise, bandaging, and infection prevention and management. Keeping a proper body weight and physical lymphatic drainage are also considered likely effective. Physical arm exercise, prophylactic antimicrobial therapy for recurrent infections, and surgical corrections can offer benefits.

According to the study of Losco et al. [12] lymphatic surgery for the treatment of lymphedema is an established approach and could improve the patients' QoL, especially in the patients who had long‐standing nonresponsiveness to conservative treatment.

Many studies have not established the effectiveness of hyperbaric oxygen (HBO) treatment for lymphedema following irradiation [11, 13].

HBO treatment involves breathing oxygen in a pressurized atmospheric setting. It is typically conducted in a chamber where the pressure is increased by greater than 1 atm absolute (ATA). Increasing the oxygen tension to hypoxic tissues results in hyperoxygenation of the blood and neovascularization. They have been associated with improved wound healing. The side effects of HBO therapy are generally transient and can often be prevented with proper screening. Patients may experience fatigue and lightheadedness after treatment. A commonly reported side effect relevant to increased pressure is middle ear barotrauma, which is generally subtle and resolves on its own. Pulmonary barotrauma is rare and can be prevented with a proper assessment before HBO treatment. Other potential side effects include mild sinus discomfort, and in rare cases, pulmonary oxygen toxicity [14, 15, 16].

Due to the nature of radiation injury, which manifests as vascular obliteration and stromal fibrosis, there is a subsequent insufficient oxygen supply to sustain normal function. The action of HBO treatment is increasing oxygen tension to hypoxic tissues, which results in hyperoxygenation of the blood and neovascularization. For many decades, it has been used as a complementary treatment for delayed–onset radiation injuries, including soft tissue and bone necrosis. This treatment may be effective in repairing damaged tissue and reducing lymphedema caused by RT [14, 15, 16]. However, there is currently no consistent data confirming the benefits of HBO therapy.

The rationale behind using HBO therapy for long‐standing arm lymphedema is based on its potential to address several underlying issues associated with chronic lymphedema. This condition, which often results from the accumulation of lymphatic fluid in the arm, can lead to connective tissue fibrosis and persistent swelling. Arm lymphedema patients who have persisted for more than 2 years often became resistant to standard treatments. We hypothesize that elevated oxygen levels can enhance cellular metabolism, diminish fibrotic changes, and improve lymphatic drainage, potentially leading to a reduction in arm lymphedema. Most recent studies [17, 18] included patients with relatively recent arm lymphedema, specifically those who had experienced the condition for 12–13 months following prior surgery.

This prospective case series aimed to study the immediate and prolonged efficacy of HBO treatment in breast cancer patients who had long‐standing (more than 2 years) lymphedema. The primary endpoint was the objective assessment (by arm volume calculation) after HBO treatment. The secondary endpoint was the subjective functional assessment of the upper limb using the Thai Quick‐DASH score: A shortened version of the Disabilities of the Arm, Shoulder, and Hand [19] after HBO treatment.

## 2 Case

Patients were selected from the medical records of the Division of Radiation Oncology at the Faculty of Medicine, Chiang Mai University, covering the period from January 1986 to December 2015. A total of 28 individuals were identified with documented cases of grades 1–2 lymphedema. After applying the exclusion criteria, only 10 patients were invited to participate in the study. Among them, six agreed to take part.

We called up breast cancer patients who fulfilled these criteria: breast cancer patients who developed persistent same‐sided arm lymphedema after postoperative RT for at least 2 years, aged more than 18 years, and were in good condition both physically and psychologically intended for HBO treatment.

Patients who had evidence of cancer recurrence, axillary mass, or venous obstruction on ultrasonography and received bilateral axillary surgery or RT were excluded. Other exclusion criteria included uncontrolled seizures, recent or ongoing surgery, infection or inflammation of the ear or sinus, severe claustrophobia, a history of chronic lung disease or emphysema, documented relative hypoglycemia in diabetic patients, or active congestive heart failure.

Each patient's medical records were examined, documenting details such as age, BMI, cancer clinical stage, time since diagnosis, type of surgery, radiation technique, radiation dose, duration and characteristics of lymphedema, and the lymphedema stage as described by the International Society of Lymphology [20]. Patients were encouraged to continue using compression clothing stockings or other techniques if they were part of their routine. Guidance on self‐care for lymphedema and the proper application of compression bandages or clothing was also provided.

All patients read and signed the consent form prior to HBO treatment, arm circumference measurement, and Thai Quick‐DASH questionnaire. This prospective case series was permitted by the Faculty of Medicine, Chiang Mai University Ethical Committee (Study code RAD‐2560‐04934).

### 2.1 Work‐Up Before HBOT

Chest radiographs were performed to evaluate chronic lung diseases. Ultrasonography of the affected and contralateral axillae, arms, and forearms was performed to exclude venous obstruction and axillary masses. Axillary scans were performed at the supraclavicular, infraclavicular, and apical regions using the pectoralis minor muscle as an anatomical reference.

### 2.2 The Arm Volume Assessment

We measured the circumference of both arms and calculated the arm volume at five different time points: before starting HBO treatment (baseline), upon completion of treatment, and at 3, 6, and 24 months post‐HBO. The arm volume was determined using the frustum formula by Casley‐Smith [21]. We measured the circumference every 4 cm along the arm, beginning at the wrist, with a total of 14 to 16 measurements per arm, depending on the distance from wrist to arm.

All patients were allowed to wear their regular compression garments during HBO sessions and afterward. However, they were instructed not to wear them on the day of follow‐up to allow for accurate measurements. To prevent inaccurate measurements, the same physician conducted all measurements throughout the study.

### 2.3 Functional Assessment

We assessed the QoL related to upper limbs using the Thai Quick‐DASH [19, 22]. It is a shortened edition questionnaire of the Disabilities of the Arm, Shoulder, and Hand (DASH), adapted for Thai speakers. This self‐evaluated questionnaire determines the upper extremity disability by asking individuals to rate their ability to perform tasks and the severity of their symptoms on a 5‐point scale. Scores range from 0 to 100, with higher scores indicating more functional limitations or disability. The Thai version was developed through cross‐cultural translation and has been validated [22].

### 2.4 HBO Treatment

All patients received HBO treatment in an HBO chamber, breathing 100% oxygen at 2.0 ATA for 90 min, five sessions per week for an overall of 30 sessions [23, 24, 25].

### 2.5 Data Analysis

Categorical variables were displayed as simple frequencies or percentages, whereas continuous variables were displayed as medians and the interquartile ranges (IQRs) due to their abnormal distribution. We used the Wilcoxon matched‐pairs signed‐rank test to determine the statistically significant changes in arm volume and Thai Quick‐DASH scores at each time point, compared to baseline measurements before HBO treatment. All analyses were conducted using STATA 17.

## 3 Results

The baseline characteristics of all six eligible patients are shown in Table 1. All the participants received postoperative RT between 1986 and 2015. The median (IQR) age at the study enrolling time was 66.5 years (62.5–69.8). Each of them had locally advanced breast cancer. Left‐sided breast cancer was the most common in our case series. All of them underwent all three treatment modalities, including surgery, RT, and chemotherapy. Five patients underwent modified radical mastectomy (MRM), while one patient had breast‐conserving surgery. Axillary lymphadenectomy and regional nodal irradiation were performed on all patients. Five of them received supraclavicular (SPC) irradiation and one patient had SPC and whole axilla irradiation. The median (IQR) duration from the onset of lymphedema as informed by the patients to HBO treatment was 10.0 years (6.5–10.0). Most of them had lymphedema stage 2 before HBO treatment. All patients had used elastic bandages for lymphedema management before participating in this study.

**TABLE 1:** Baseline characteristics.

Patient no	Age	Stage	Type of surgery	Positive LN/total LN	Radiation technique	Radiation field	RT dose (Gy/fraction)	Time to development of lymphedema after RT (years)	Duration from onset lymphedema to HBOT (years)	Lymphedema stage [21]	Previous treatment of lymphedema
1	69	IIIA	MRM	0/17 Post NAC	2D‐RT	CW + SPC	50/25	4	14	2	Elastic bandage
2	64	IIB	Lumpectomy with AXLND	0/23 Post NAC	2D‐RT	Breast + SPC	60/30	6	3	2	Elastic bandage
3	73	IIB	MRM	0/17	2D‐RT	CW + SPC	50/25	5	10	2	Elastic bandage
4	61	IIB	MRM	0/11	2D‐RT	CW + SPC	50/25	1	17	2	Elastic bandage
5	81	IIA	MRM	2/6	2D‐RT	CW + SPC	50/25	22	10	2	Elastic bandage
6	61	IIIA	MRM	4/6 with ECE	IMRT	CW + SPC + whole axilla	50/25	1	2	1	Elastic bandage

Abbreviations: 2D‐RT = two‐dimensional radiotherapy, AXLND = axillary lymph node dissection, CW = chest wall, ECE = extracapsular extension, HBOT = hyperbaric oxygen therapy, MRM = modified radical mastectomy, NAC = neoadjuvant chemotherapy, RT = radiotherapy, SPC = supraclavicular.

Both subjective and objective assessments of lymphedema from the baseline to each time point after HBO treatment for each patient are shown in Table 2. Patient number 6 did not attend the follow‐up at 24 months after HBO treatment due to moving to another province. As shown in Table 3, the arm volume decreased compared to baseline at 3, 6, and 24 months after HBO, with median changes of 28, 87, and 51 cm^3^, respectively. However, these changes were not statistically significant. The mean Thai Quick‐DASH scores of patients decreased at each time after treatment compared to the baseline: 10.0 in the 3rd month, 4.4 in the 6th month, and 4.4 in the 24th month. Despite this decrease, there was no statistically significant difference. All six patients experienced no complications during HBO treatment.

**TABLE 2:** Subjective and objective assessment of lymphedema from baseline to every time points after HBOT.

Arm volume by circumferential measurement (cm^3^)	Patient no. 1	Patient no. 2	Patient no. 3	Patient no. 4	Patient no. 5	Patient no. 6	Total
Baseline (*n* = 6)	2445	2948	3816	2346	4807	3277	3112 (2445–3816)
Complete treatment (*n* = 6)	2378	3035	3875	2437	5005	3115	3075 (2437–3875)
3 months^a^(*n* = 6)	2530	2786	3775	2330	4901	3047	2916 (2530–3775)
6 months^a^(*n* = 6)	1892	2875	3685	2384	5269	3176	3026 (2384–3685)
24 months^a^(*n* = 5)	1903	2885	3765	2336	5002		2885 (2336–3765)

Quick‐DASH score							
Baseline (*n* = 5)	10.72		25	25	11.36	29.54	25.00 (11.36–25.00)
Complete treatment (*n* = 5)	10.62		22.73	25	4.5	18.18	18.18 (10.62–22.73)
3 months^a^(*n* = 5)	13.74		11.36	15	4.5	11.36	11.36 (11.36–13.74)
6 months^a^(*n* = 4)	8.78		6.8	27.5	4.5		7.79 (5.65–18.14)
24 months^a^(*n* = 4)	8.78		11.36	25	4.5		8.78 (4.50–11.36)

^a^ Months after complete treatment.

**TABLE 3:** Changes in arm volume by circumferential measurement and Quick‐Dash scores in six patients after treatment compared to baseline.

Time period	Arm volume (cm^3^) median (IQR) (*p* ^a^)	Quick‐DASH score median (IQR) (*p* ^a^)
Complete treatment to baseline (*n* = 5)	73 (−67 to 91) (*p* = 0.463)	−2.27 (−6.86 to −0.10) (*p* = 0.057)
3 months after complete treatment to baseline (*n* = 5)	−28 (−162 to 85) (*p* = 0.463)	−10.00 (−13.64 to −6.86) (*p* = 0.080)
6 months after complete treatment to baseline (*n* = 4)	−87 (−131 to 38) (*p* = 0.345)	−4.40 (−12.53 to 0.28) (*p* = 0.273)
24 months after complete treatment to baseline (*n* = 4)	−51 (−63 to −10) (*p* = 0.345)	−4.40 (−10.25 to −0.97) (*p* = 0.095)

^a^ Wilcoxon matched‐pairs signed‐rank test.

## 4 Discussion

HBO treatment has the potential to improve lymphatic drainage by increasing tissue oxygenation, promoting neovascularization, and reducing vascular stromal fibrosis, but it has shown mixed results, as demonstrated by our study. This finding is consistent with several prior studies [11, 13, 23], which also found limited evidence supporting HBO's use in managing arm lymphedema. These contrasting findings in the literature highlight the variability in responses to HBO. Additionally, a recent systematic review [26] reported improved QoL in various conditions, such as postconcussion syndrome and diabetic ulcers, but found no evidence of QoL improvement in patients with chronic arm lymphedema. This discrepancy underscores the need for further exploration into why HBO may benefit some conditions but not others.

Some studies have demonstrated specific benefits for lymphedema. Pritchard et al. [24] found that two patients with severe lymphedema experienced marked and sustained reductions in arm volume for 12 months following HBO treatment, suggesting that certain patients may benefit from this therapy. Similarly, Gothard et al. [23] observed a significant reduction in arm volume in breast cancer patients with lymphedema, although there were no corresponding improvements in QoL, highlighting a disconnect between objective and subjective outcomes.

Conversely, Teas et al. [25] and Gothard et al. [27] found no significant changes in arm volume after HBO treatment, despite some patients reporting symptom relief. Teguh et al. [28] also reported improvements in pain scores and lymphedema‐related complaints, indicating that while HBO may not consistently reduce arm volume, it could still alleviate symptoms, which is crucial for enhancing patient comfort and well‐being. A Danish randomized controlled study [18] found that while HBO improved QoL in lymphedema patients 1‐year postsurgery, it did not significantly affect arm mass or volume. This contrasts with some studies showing volume reduction, emphasizing the variability in individual responses and the need for further research to identify which patients may benefit most from HBO therapy.

The complexity of lymphedema progression may also explain these mixed results. In our cohort, the median time for lymphedema development was 10 years postradiation, with both early‐ and late‐onset cases observed. A large cohort study suggested that delayed‐onset lymphedema is often influenced by factors such as infection, trauma, and weight gain. Furthermore, a recent prospective study on patients with more recent lymphedema reported significant improvement following short‐course HBO treatment [29], indicating that the timing of intervention may play a critical role in treatment efficacy.

In our case series, HBO was well‐tolerated, with no side effects reported, supporting its safety. Most patients had high BMIs and underwent MRM with axillary lymph node removal, both risk factors for lymphedema [7]. Paiva et al. [30] confirmed the direct relationship between lymph node dissection and lymphedema, with our patients averaging 13 nodes removed. Irradiation to the supraclavicular and axillary regions also increased lymphedema risk by 3.57 times [31].

We utilized circumferential arm volume measurements and Quick‐DASH scores for upper extremity disability, both recommended by the National Lymphedema Network [32] to capture the physical and functional changes post‐HBO treatments. However, these tools are limited by their lack of sensitivity, potential for measurement variability, and inability to assess underlying tissue changes.

Novel tools such as bioimpedance spectroscopy (BIS) [33], tissue dielectric constant (TDC) [34], magnetic resonance imaging (MRI), near‐infrared fluorescence imaging (NIRF), and lymphoscintigraphy are gaining traction for their ability to detect subtle fluid changes and assess tissue composition. BIS provides an objective analysis of extracellular fluid volume, which is critical for the early detection of lymphedema, while TDC offers a noninvasive method to assess localized tissue water content. Novel imaging tools offer detailed insights into the structure and function of the lymphatic system, aiding in early detection and more accurate assessment of lymphedema. These technologies, alongside traditional methods, enable more accurate and earlier diagnosis of lymphedema progression and treatment monitoring, which were unavailable in our clinic.

This is the main limitation of our study. Other limitations include the small sample size (*n* = 6), which reduced the statistical power needed to detect significant changes. Additionally, the wide variation in lymphedema duration before HBO treatment (ranging from 2 to 17 years) may have contributed to patient variability. The absence of a control group also makes it difficult to attribute changes solely to HBO, as they could be influenced by natural disease progression or other interventions.

## 5 Conclusions

Although HBO treatment appeared to reduce both the mean arm volume and Quick‐DASH score in chronic lymphedema in this patient series, these changes were not statistically significant.

Future research should prioritize larger, randomized controlled trials to more accurately assess the efficacy of HBO therapy for lymphedema in breast cancer patients. These studies should stratify patients based on the duration of lymphedema to evaluate how the timing of intervention affects outcomes. Additionally, investigating biomarkers or advanced imaging techniques could help elucidate the mechanisms behind HBO's effects on lymphatic function. Extending follow‐up beyond 24 months would be beneficial to determine whether the observed benefits are sustained long term and assess the risk of relapse.

## Author Contributions

**Imjai Chitapanarux:** conceptualization, writing – original draft, review and editing, supervision. **Siriarrayapa Chachvarat:** investigation, writing – original draft. **Patumrat Sripan:** statistical analysis. **Thienchai Pattarasakulchai:** investigation. **Nuttaya Pattamapaspong:** writing – review and editing. **Thanat Kanthawang:** writing – review and editing. **Montana Buntragulpoontawee:** writing – review and editing.

## Ethics Statement

This study was approved by the Research Ethics Committee of the Faculty of Medicine, Chiang Mai University.

## Conflicts of Interest

The authors declare no conflicts of interest.

## Data Availability

The datasets used and/or analyzed during the current study are available from the corresponding author upon reasonable request.
